# Gender differences in type 2 diabetes treatment and management: a qualitative study in an urban slum population from Dhaka, Bangladesh

**DOI:** 10.1186/s12939-025-02611-2

**Published:** 2025-09-30

**Authors:** Ruchira Tabassum Naved, Aloka Talukder, K. M. Thouhidur Rahman, Sohel Choudhury, Rajiv Chowdhury, John Danesh, Emanuele Di Angelantonio, Md Khalequzzaman, Md Alfazal Khan, Simon Griffin, Nick Mascie-Taylor

**Affiliations:** 1 Gender, Equity and Rights, Maternal and Child Health Division, icddr,b, Dhaka, Bangladesh; 2 Department of Public Health and Informatics, Bangabandhu Sheikh Mujib Medical University, Dhaka, Bangladesh; 3 National Heart Foundation Hospital and Research Institute, Dhaka, Bangladesh; 4 Stempel College of Public Health and Social Work, Florida International University, Miami, FL USA; 5 British Heart Foundation Cardiovascular Epidemiology Unit, Department of Public Health and Primary Care, University of Cambridge, Cambridge, UK; 6 Health Systems and Population Studies Division, icddr,b, Dhaka, Bangladesh; 7 Department of Public Health and Primary Care, University of Cambridge School of Clinical Medicine, Cambridge, UK

**Keywords:** Diabetes, Type 2 diabetes, Treatment and management, Urban slum, Bangladesh, Gender

## Abstract

**Background:**

More than 80 million South Asians are living with type 2 diabetes (T2D). Despite increasing recognition that gender contributes to the prevention, treatment and outcomes of diseases, studies on gender and diabetes are scarce globally and particularly in impoverished and typically patriarchal settings, where gender hierarchies might be more pronounced. We explored how differences in gender roles, gendered access to, and control over resources, and social norms contribute to gender differences in treatment and management of T2D in an urban slum of Bangladesh.

**Methods:**

Data were collected between January and April, 2021 from Bauniabadh slum in Dhaka city, Bangladesh using 8 Key Informant Interviews (KII) and 60 In-Depth Interviews (IDI), with equal numbers of women and men aged 35 years or more, and a confirmed diagnosis of T2D at least two years prior to the current study. Within and across gender analyses were performed. We used positive and negative deviance approaches to understand the factors underlying variation in treatment and management of T2D.

**Results:**

Individual, household and structural factors posed gendered challenges to management of T2D. Compared to men, women were more prone to financial and time constraints originating from rigid gender roles and discriminatory entitlements in the household and society. Women employed more coping strategies than men to deal with financial constraints when seeking medical treatment. Strong household financial status, relevant connections and support from the family underlied high-performance by men in the management of T2D. Household financial status was a necessary, but not sufficient condition for high-performance among women. In addition, women’s economic empowerment, agency, voice and power appear to be key for optimising T2D management.

**Conclusions:**

The findings highlight the importance of tailored programmes and policies for T2D to reduce gender inequities T2D management.

**Supplementary Information:**

The online version contains supplementary material available at 10.1186/s12939-025-02611-2.

## Background

South Asia is one of the major epicentres of the global epidemic of non-communicable diseases (NCDs) such as type 2 diabetes [1] (henceforth referred to as diabetes). Despite the fact that diabetes is largely preventable [2], currently, 87 million South East Asians are living with the condition [3], including 10% of adults in Bangladesh [4, 5].

There are increasing indications that gender differences might be important in the prevention, treatment and outcomes of NCDs [6–8]. Gender disparities are common in attitudes towards disease prevention, treatment and management. These arise from sociocultural structures and processes, such as gender hierarchy, socially defined gender norms that accord men and women different roles and power, risk-taking behaviours, different nutrient intakes, lifestyles, and stress [4, 9–11]. For example, men have access to more varied sources of information about diabetes than women (such as the internet and television) [12]. A systematic literature review on gender differences in access to diabetes care found that the major impediments for service uptake for women were lack of finances, lack of time due to their caregiving role and the associated work schedule, denial of disease status and severity, and lack of agency [13]. However, service uptake varies by setting. It was less regular for US women than men [14], higher among women in Israel [15] and lower amon South Asian women due to restricted mobility and autonomy [16].

Suboptimal management of diabetes is also driven by socialisation, that encourages women to be more altruistic than men and to prioritize others’ needs over their own [17]. This is manifested in an unwillingness to change their family’s lifestyle to accommodate their own health needs [17, 18]. Moreover, women may fear lack of support from their families and consequently may be unwilling to disclose their illness and related needs [19]. Women may also not find adequate time for self-care [20] including adherence to recommendations concerning diet and physical activity due to factors such as domestic responsibilities and neighbourhood characteristics such as safety, lighting and traffic density which have differential impact by gender [21–28]. Current literature on gender and diabetes is, however, scanty and inconclusive.

Gender differences in diabetes treatment and management are likely to be most pronounced in settings with high gender asymmetry such as Bangladesh. Bangladesh has a patriarchal social structures. Social norms dictate highly skewed gender relations in favour of men supported by gender-biased perceptions and attitudes, values, systems, laws (e.g., inheritance) and other social structures. Such gender asymmetry is reflected in the perception of lineage, son preference, low investment in females, patrilocal marriages, village exogamy, and low social status and position of females relative to males. Females face numerous restrictions and males are responsible for keeping females ‘in line’ [29–31]. Restrictions are imposed on the mobility of females [32]. They have to observe *purdah* and avoid interaction with males. Women usually have minimal decision-making powers in the household [33, 34]. Any transgression from prescribed gender roles is met with attempts at disciplining women using verbal and other forms of abuse [30, 35].

An interaction between gender and poverty may compound the challenges around treatment and management of diabetes. Slums in Bangladesh accommodate a large impoverished population. In slums of Bangladesh, 99% males and 36% females are employed [36]. Both sexes pursue relatively low paid jobs. The men from Dhaka slums usually work as day labourers, service holders, and engage in small business and rickshaw driving. The women work mainly as garment factory workers, domestic help, and day labourers [37]. Average household monthly expenditure is USD153 in Dhaka slums. Of which 53% is spent on food. Around 78% slum households in Dhaka cannot save [37].

In slums of Bangladesh, 55% of households live in one room [36]. Cooking facilities, toilets and source of drinking water are commonly shared in Dhaka slums [37]. Gas is used by the majority of households for cooking. Only 16% of slum households in Dhaka possess a refrigerator [37]. Such poor living conditions and resource constraints are likely to introduce fierce competition for household resources and intensify gender differences in treatment and management of diabetes.

Although an understanding of different challenges and needs of women and men, and particularly of those living in poverty in under-served urban areas, is critical for designing appropriate and effective patient-centred interventions and services, to our knowledge, there has been no study addressing this issue in Bangladesh. We aimed to explore how differences in gender roles, gendered access to and control over resources and social norms contribute to the management of diabetes in a poor urban setting (slum) in Dhaka, Bangladesh.

## Methods

This qualitative study was embedded within an on-going prospective cohort study the “Bangladesh Longitudinal Investigation of Emerging Vascular and nonvascular Events (BELIEVE)”. This study has been described elsewhere [38]. Briefly, the BELIEVE study was funded by UK Research and Innovation (UKRI) and set up by the University of Cambridge, (i.e., the coordinating centre), University College London and Aberdeen University, UK with four national collaborating centres in Bangladesh: the National Heart Foundation (NHF) and Research Institute; Bangabandhu Sheikh Mujib Medical University (BSMMU); the Institute of Epidemiology, Disease Control and Research (IEDCR); and the International Centre for Diarrhoeal Disease Research, Bangladesh (icddr,b). The BELIEVE multi-site cohort study (*N* = 73,883) is designed to quantify the burden of non-communicable diseases (NCDs) in urban, rural, and urban-slum areas of Bangladesh and provide insight into their demographic, societal, economic, behavioural and genetic determinants.

### Study setting, sample selection and data collection

The present qualitative study was conducted in the urban slum area of Mirpur in Dhaka city, known as Bauniabadh. Data were collected using eight Key Informant Interviews (KIIs) and 60 In-Depth Interviews (IDIs). The key informants were selected purposively from among the health care providers serving the selected slum in order to cover the types of providers considered important in the studied slum. They were identified with the help of community leaders/influential slum dwellers and in-depth interview (IDI) participants. We conducted IDIs with 30 men and 30 women from a randomly selected sample of 90 men and 90 women aged > = 35 years living with diabetes for 2 or more years from the BELIEVE-Slum population (*N* = 5,332), who were matched by education. We stopped participant recruitment once we had successfully interviewed 30 men and 30 women.

Due to high rates of COVID-19 in the research setting at the time of this study, interviews were conducted by telephone. Interview guides included questions on knowledge about diabetes care and management; adherence to required treatment and management guidelines; and challenges experienced (see Supplementary Material 1).

Two female and three male interviewers with Masters degrees in Anthropology collected the data. The team received training on gender, qualitative research methods and study guides. The interviews were gender-matched. Daily de-briefing meetings were held during fieldwork for sharing findings and to discuss any problems and potential solutions. Data were collected between January and April, 2021.

### Ethical considerations

Potential study participants were orally informed of the purpose and nature of the study, its expected risks and benefits, the voluntary nature of participation, and confidentiality. Oral consent was recorded. We requested the study participants to ensure that the interview could be undertaken in private and non- use of a loudspeaker during the interview. In the event of any interruption the interview was re-started via a new phone call. Interviews were digitally recorded anonymously. We provided participants with a one-time electronic money transfer of BDT 100 (about 1 USD). We stored the names and addresses of the informants in a separate file for follow-up phone interviews. Pseudonyms and unique numbers were used for identifying the informants. We stored the data on a password protected computer with access limited to the researchers. Recordings were destroyed upon completion of the study. We analysed de-identified data, the results presented in this paper are all anonymised. The study received ethical approval from icddr, b’s Institutional Review board (PR-19128).

### Data management, processing and analysis

All interviews were transcribed verbatim. The researchers examined accuracy and completeness of the transcripts by checking a random sample of 20% of IDIs and KIIs and corrections were made where necessary. The data were coded by three researchers using Atlas ti [39]. Inter-coder reliability was ensured by following a rigorous training process for standardisation of coding and cross-checking. Coded data were analysed by the lead author. The focus of the analysis was the pre-COVID-19 pandemic period. We used narrative analysis to examine each transcript for a core narrative or ‘story’ of a man or a woman about the diagnosis of diabetes and its management [40, 41]. We compared these core narratives across participants to identify distinct and shared features of the narratives. We assessed the adherence of the patients to treatment recommendations in five domains: (1) medical treatment; (2) adherence to recommended medication in terms of both dose and frequency; (3) starch intake; (4) sugar intake; and (5) physical activity. Adherence was coded as: (1) Regularly or almost regularly; (2) Intermittently; and (3) Never or a few times. Regularly or almost regularly accessing a medical doctor for treatment of diabetes was defined as visiting once every two to three months. Those, who regularly or almost regularly followed the recommendations in 4–5 domains (e.g., follow-up visit to medical doctos, taking recommended medications, adherence to dietary recommendations regarding starch intake and eliminating sugar consumption, doing regular physical activity) were labelled as ‘high-performing’, those who followed the recommendations only once/few times or never in 3 or more domains were considered as ‘low-performing’ and the remainder were classified as ‘medians’. We examined and compared the stories of patients in the 3 different adherence categories in order to tease out the factors underlying their responses.

## Results

### Characteristics of the in-depth interview participants

Most of the IDI participants, who were all Muslims, were aged 49 or older (*n* = 38, Table 1) with an equal distribution of men and women in this age group. Among the rest of the sample women were younger than the men. Thirty-four participants had been diagnosed with diabetes for 5–9 years. Overall, the level of education was low in the sample with one in three having no education and one-third having only primary education. Women had formal education than men. Only five out of 30 women were employed at the time of the study, compared to 28 out of 30 men. Nine women had never been employed, whereas all men had been ever-employed. Most of the men were pursuing small business (*n* = 13), while the others were engaged in low-paid jobs (e.g. driver, security guard). The number of women living in an extended family was slightly higher than that of the men. The reported level of household poverty was higher among women than men.

**Table 1:** Characteristics of the male and female diabetes patients interviewed in-depth, Bauniabadh slum, Dhaka, 2021

Characteristic	Male (*n* = 30)	Female (*n* = 30)	Total (*N* = 60)
Age group, years			
35–39	1	4	5
40–44	2	4	6
45–49	8	3	11
49+	19	19	38
Duration of diabetes, years			
3–4	6	2	8
5–9	15	19	34
9+	9	9	18
Highest level of education attained, years			
0	8	13	21
1–4	5	3	8
5–9	9	11	20
10	4	2	6
10+	1	0	1
Missing	3	1	4
Employment status			
Currently employed	28	5	33
Ever-employed previously only	2	16	18
Never-employed	0	9	9
Religion			
Islam	30	30	60
NGO affiliation			
Yes	1	4	5
No	17	26	43
Missing	12	0	12
Family structure			
Extended	17	20	37
Nuclear	13	10	23
Socio-economic Status			
Poor	10	18	28
Non-poor	20	12	32

### Treatment-seeking

Most of the study participants were aware of more than one service provider for treatment of diabetes. However, the sources of this knowledge differed between women and men, with greater diversity among men. Neighbours and relatives almost exclusively served as the source of this knowledge for women. The additional sources of knowledge for men were exposure to signboards and the internet.

Table 2 presents information on participant treatment-seeking and adherence to recommendations. With the exception of one woman and one man who used a traditional remedy, all participants sought allopathic treatment at least once. The overwhelming majority of participants (50 out of 60) did not access medical treatment regularly. Sixteen men and 14 women accessed them only initially, while 8 men and 10 women accessed them intermittently. Across gender, a large proportion of participants consulted the pharmacy counter staff mainly for monitoring glucose levels and sometimes for advice regarding medication and life style.

**Table 2:** Provider accessed and compliance to diabetic treatment by gender, Bauniabadh slum, Dhaka, 2021

	Male (*n* = 30)	Female (*n* = 30)	Total (*N* = 60)
Medical doctors accessed			
Medical doctors			
Regularly or almost regularly	5	5	10
Intermittently	8	10	18
Never/a few times	17	15	32
Adhered to recommended medications			
Regularly or almost regularly	15	12	27
Intermittently	12	15	27
Never/a few times	2	3	5
Missing	1	0	1
Adhered to dietary recommendations regarding starch intake			
Regularly or almost regularly	14	18	32
Intermittently	11	5	16
Never/a few times	4	6	10
Missing	1	1	2
Eliminated sugar consumption			
Regularly or almost regularly	10	17	27
Intermittently	16	5	21
Never	0	1	1
Missing	4	7	11
Undertook physical activity			
Regularly or almost regularly	9	10	19
Intermittently	14	13	27
Never/a few times	7	7	14

As shown in Table 3, many participants faced challenges in regularly seeking treatment. These challenges clustered into two primary groups, namely, (1) financial constraint; and (2) gender issues.

**Table 3:** Challenges in treatment seeking by gender (multiple choice), Bauniabadh slum, dhaka, 2021

	Male (*n* = 30)	Female (*n* = 30)	Total (*N* = 60)
Challenges in treatment seeking	17	23	40
Financial constraint	16	21	37
Time constraint	2	3	5
Gender issues	2	11	13
Lack of education/agency	0	7	7
Prioritizing needs and well-being of children/family	0	4	4
Concern re Purdah	0	1	1
Inability to attach importance	2	0	2
Structural/social issue: Distance/traffic congestion	2	7	9
Gender issues related to service quality	0	4	4
Dishonesty of doctors	0	1	1
Lack of empathy & listening skills/psychological violence by the provider	0	3	3
Absence of female doctor	0	2	2
Timing of service	1	0	1
Challenges in complying to recommended medication	17	16	33
Financial constraint	15	16	31
Negligence/forgetfulness	2	0	2
Challenges in complying to recommended diet	13	12	25
Lack of self-control in reducing starch/rice intake	8	3	11
Making bread on top of cooking rice for the family due to increased workload	1	6	7
Lack of self-control in cutting sugar intake	4	4	8
Financial challenges in buying vegetables for substituting rice	1	2	3
Prioritising family members’ needs compromising proper diet	1	2	3
Inadequate and untimely gas supply	2	3	5
Inability to attach importance	2	0	2
Challenges in complying to recommended physical activity	19	24	43
Comorbidities	9	13	22
Visible gender issues	11	18	29
Time constraint due to responsibilities unrelated to the household	11	6	17
Time constraint due to household chores/responsibilities	0	11	11
Structural issues	2	13	15
Lack of space for walking	2	7	9
Heavy traffic, road maintenance work and safety	1	7	8
Conflict with the timing of cooking gas supply	0	4	4
Gender roles/harassment	0	4	4
Laughed at by men while walking briskly	0	1	1
Harassment/Sexual remarks	0	3	3
Criticism and social unacceptance	0	3	3
Other	6	2	8
Conflict with sleep, laziness, negligence	4	2	6
Covid-19 related restrictions	2	0	2
Interruption due to meeting acquaintances on the street	1	0	1

Across gender, the main challenge in medical treatment-seeking was financial, experienced more often by women then men (21 women vs. 16 men) (Table 3). Financial constraints were more pronounced during the COVID-19 pandemic. Both IDI and KII data revealed that, compared to the pre-pandemic period, participants were much more likely to seek counter advice from pharmacies regarding diabetes treatment and management during the pandemic, as it did not cost any money and the pharmacies were closer.

Women were more vulnerable to financial constraints than men as most women did not have their own income; had inadequate access to and control over household resources; lacked voice and were altruistic (Tables 3 and 5). Non-working women were in a more disadvantaged position with respect to seeking treatment, not only compared to men, but also to their working peers.

“Compared to working women, women, who do not earn an income are less likely to seek treatment. The husband considers the household needs before spending money on her treatment.”

-KII 2, female

Despite greater time constraint, the working women seemed better at accessing medical doctors compared to their non-working peers. Their work related mobility and greater ease of interaction with the world outside home may have contributed to this. However, it must be noted that treatment-seeking of working women was also compromised in some cases as it largely depended on the size of their income and household needs. Even if a woman had some money, household needs competed with treatment, and she tended to prioritise the former, particularly children’s well-being, over her own treatment. As demonstrated in Case Study 1 (Table 5), household poverty, lack of access to, and control over household resources, violence, competing priorities and altruism often made it impossible for a woman to meaningfully access treatment.

The data indicate that despite greater time constraint younger women aged 35–49 years tended to access medical doctors more than the older women. Higher education and agency of the younger women may have helped them to do so. Participants employed both positive and negative coping strategies to deal with financial constraints in treatment-seeking (Table 4). The positive coping strategies included: finding less expensive facilities/providers; revealing financial difficulties to the provider and obtaining treatment at a reduced price or free of charge, or receiving support from diverse sources. Overall, women used more positive coping strategies compared to men when seeking treatment. It seems that more women disclosed their financial problems to providers and drew on financial support in seeking medical treatment compared to men.

**Table 4:** Coping with financial constraints in treatment seeking and obtaining medicines, Bauniabadh slum, Dhaka, 2021

	Male (*n* = 30)	Female (*n* = 30)	Total (*n* = 60)
Coping with financial constraints to seek medical treatment			
Positive coping strategies	1	4	5
Revealing financial challenges to the provider and obtaining support from diverse sources	1	3	4
Choosing less expensive facility/provider	1	1	2
Negative coping strategies	6	13	19
Skipping/stopping follow-up visits	3	8	11
Seeking advice from pharmacists/other diabetic patients	3	7	10
Taking traditional/home remedy	0	1	1
Coping with financial constraints in obtaining medicines			
Positive coping strategies	3	8	11
Obtaining cheaper/free medicine/Medicine on credit	2	2	4
Getting support from other sources/Saving from daily expenses	0	6	6
Switching to tablet from insulin consulting the doctor	1	0	1
Negative coping strategies	2	5	7
Switching to tablet from insulin without consulting a doctor	1	1	1
Traditional/home remedy	1	1	2
Reducing dose on own/stopping medicine intake during financial crisis/taking medicine only when seriously ill	2	6	8

Negative strategies in coping with financial constraints in treatment seeking included: not seeking medical treatment/skipping or stopping follow-up visits; seeking advice from the pharmacists/less optimal providers nearby/other diabetic patients; and using a home remedy. More women were found to use negative coping strategies than men (13 vs. 6).

*“I got my blood tested and …further investigations were advised. [The investigations] required about tk. 3000 (USD 35). So*,* I left that facility and went to a public hospital. …I told them [the providers] that I have no money to get the investigations done. [The provider] then prescribed me insulin and asked me to get the investigations done as soon as I can arrange the money.*

*…At times I go to the pharmacy for measuring my glucose level paying tk. 30 (USD 0.35). When the glucose level increases the pharmacist suggests me to increase the dose of insulin and advises me to reduce it*,* when it is low. I follow his advice. I could not undergo the investigations yet. I feel embarrassed asking for money from my sons-in-law.” *

*-IDI 9, female*

A cluster of challenges were relevant exclusively to female patients while seeking treatment (Table 3). These were: lack of education/agency (*n* = 7); altruism expressed in prioritising needs and well-being of the family over her own treatment (*n* = 4); and issues related to service quality (*n* = 4). Distance to the provider was more of a problem for women than men (7 vs. 2). Some of these issues were interlinked and interactions between them often compounded the hurdles in accessing treatment. For instance, distance to the provider posed a multi-faceted challenge to the women. It overlapped with challenges of finances, time availability, women’s agency, and some structural issues (e.g., traffic congestion). Distance implied extra costs for travel. Financial resources were scarce for women not only because of household poverty, but also because most of them did not earn any money and did not have adequate access to or/and control over household financial resources. Women often lacked the time to go to the provider, due to household responsibilities. Lack of agency of women compounded these challenges. Rigidly defined gender roles confining women to the house made many of them lack confidence in moving about outside the home. Some of them feared getting lost and not being able to deal appropriately with the outside world. Thus, for a few female patients, treatment-seeking was dependent on whether they could find a chaperone. Data show that finding one could be a challenge. Their husbands, if alive, often had compromised health, which meant they were unable to accompany the women. Their children, on the other hand, were likely to be busy either with education or earning a living.

“At times I can’t attend the [scheduled] follow-ups since I cannot go alone. …I cannot go anywhere alone fearing of getting lost. I do not virtually know any of the places. …Someone has to accompany me. [My husband] cannot go with me as he is ill.”

-IDI 15, female

“Going to the hospital is a problem. Previously my son used to accompany me, but, now, he does not. …How many times can he go with me? If he is willing he’ll go, if not – he cannot be dragged into it. …So, I have to go alone, which is inconvenient. I annoy the bus conductor by repeatedly telling him that I don’t know the place and asking him whether we got there [my destination].”

-IDI 30, female

Women, often did not voice their needs and tended to put other requirements above their own (Table 5). While many men had to compromise medical treatment-seeking for the purpose of subsistence of the family, women seemed to forgo such treatment considering not only subsistence, but broader well-being of the family (e.g., children’s education).

“The cost of my treatment is tk. 5000–6000 (USD 58–70) [per month]. I can bear the expense two months at a stretch and then I have to decide whether to feed the family or to continue my treatment. This is why I often have to stop the treatment, which results in poor health. … I have not visited my doctor for over a year now.”

-IDI 8, male

Women reported not seeking treatment for: taking care of an ailing husband and other household members; not diverting resources from investments in the well-being of children/grandchildren; and not bothering other family members for the purpose of their treatment. A woman, for example, stopped going for follow-up visits ever since the pandemic started. Her husband’s business of ironing clothes was the primary source of income for the household but business was very slow during COVID-19. Alos, her daughter contributing to the household lost her job as a school teacher. The woman reasoned that under these circumstances, any extra financial burden was likely to hamper her children’s education and thus, she stopped taking treatment. She told the interviewer,

“Approximately tk.5000 (USD 58) is spent on each follow up visit. I have stopped going for follow-ups and stopped taking medicines since these expenses will interfere with coverage of my children’s education.”

-IDI 3, female

Data suggest physical and structural issues also create significant barriers to women’s treatment-seeking (Table 3). These are: distance to the facility, traffic congestion and health service quality.

“Distance [to the hospital] is an issue. There is traffic congestion on the way [to the hospital]. If I start at 6 am I arrive [at the hospital] at around 8 am. If I start at 8 am I’ll reach the hospital only around 1 pm.”

-IDI 26, female

Service quality issues that discouraged women from seeking treatment were non-cordial and abusive behaviour, lack of listening skills and empathy of the provider; a perception that unnecessary investigations and procedures were recommended for the facility’s financial gain; and lack of female providers. One male patient mentioned that the service timing was unsuitable for him. Interestingly, two men reported inability to attach importance to the condition (diabetes) as a reason for not seeking medical treatment regularly.

“It is impossible to talk to the doctor! Don’t I need to tell them what has happened to me? If I say anything s/he pounces on me!”

-IDI 23, female

### Adherence to recommended medication

Adherence to recommended medication was poor across both genders with only half of the male sample and a little less than that of the female sample regularly following the recommendations (Table 2). A few patients (2 men and 3 women) never or rarely took medication as recommended, while the rest adhered only intermittently. Financial constraints were again the main reason for not adhering to the recommended medications both both female and male patients (15 men vs. 16 women) (Table 3). More younger women tended to report financial challenges in adhering to recommended medications than the older women. Financial constraints were heightened during the pandemic compromising the capability of patients to purchase the recommended medication. A pharmacist observed,

“Due to lockdown many people cannot work. Now, where will they find money [for buying medicines], if unemployed? So, I have to sell medicines to the local people on credit. …Some are obtaining medicines on credit and some are not taking any medicines at all.”

-KII 2, male

The common positive coping strategy for adhering to medication for both men and women was obtaining cheaper or free medicines or medicines on credit (2 men and 2 women). Since insulin was much more expensive than tablets, a man switched to tablets from insulin after consulting the doctor. Six women saved money from daily expenses and used money from microcredit to buy medicines (Table 4). The negative coping strategies for dealing with lack of money to buy medicines included reducing the dose of medicine or taking medicines only when feeling very ill, or stopping medication altogether when lacking money (2 men vs. 6 women). One man and one woman used traditional/home remedies. One man and one woman reduced the costs of medication by switching from insulin without consulting a doctor. Table 5 illustrates how the same patient could use multiple coping strategies, be it positive or negative in dealing with financial constraints in buying medicines.

**Table 5:** Case Study 1: Amena's challenges in dealing with diabetes

About seven or eight years ago Amena started feeling excessively thirsty. She also had some other symptoms. She went to a private hospital and was diagnosed with diabetes. Amena does not earn any money and her husband is unwilling to cover the costs of her treatment. When the question of her treatment arises, her husband shouts at her and there is a row. He curses her and wishes she was dead so that he can remarry. Even if Amena can arrange some money she often has to sacrifice her treatment for sending gifts to her daughters or treating them when they visit. This is essential for keeping her sons-in-law happy. Under these circumstances, she can afford to get her glucose level tested at the nearby pharmacy only when she feels very unwell. She cannot take the medicines regularly for lack of money. She takes them only when she feels very sick. Previously, she could manage to buy some medicines saving money from household expenses. But during the COVID-19 pandemic her husband lost his job and no savings could be accumulated anymore. At the time of the interview, she had not been taking her medicines for the last two to three months. Even when she can afford to buy the medicines she takes one tablet per day instead of two so that they last longer.	

Forgetfulness and negligence were mentioned by two male patients, but not by any women, as deterrents to adhering to recommended medication (Table 3).

### Adherence to dietary recommendations

The key informants claimed that male patients are less likely to follow the recommended healthy diet than females, the main reasons being men’s low self-control in modifying dietary habits and inability to attach importance to the recommendations. This section, focuses mainly on adherence to recommendations to reduce starch consumption and to eliminate sugar intake. As mentioned by a female patient, the essence of being a Bengali is portrayed in the following proverb: eating rice and fish make a person Bengali macheybhatey Bangali). According to most of the IDIs patients were advised to have one cup of rice a day and to have bread (ruti) at two meals. Reducing consumption of rice to this level presented a challenge to the study population. Data show that about half of the male sample and about one third of the female sample were unable to regularly reduce rice intake and substitute it with bread (Table 2). A rice-less or low-rice meal felt unfulfilling and non-satiating to many study participants, particularly men and older women, who found it difficult to break away from the traditional diet.

“No patient with diabetes can follow the dietary recommendations. The doctor suggested eating two cups of rice. How can a patient survive on two cups of rice? Would you be able to manage the whole day with just two cups of rice?”

-IDI 9, male

“It was challenging for me to follow the doctor’s [dietary] recommendations. …. [My family] told me that I have to follow the recommendation for my survival. I used to shout and swear at them and demand more food.”

-IDI 13, male

The patients were advised to substitute starch with plenty of vegetables. Unfortunately, vegetables were more expensive than rice and a few study participants could not afford to buy adequate amounts of vegetables. Issues related to intrahousehold food distribution, disfavouring women in particular, also interfered with consuming the required amount of vegetables in place or rice.

The recommended two *ruti-*meals had obvious implications for women’s workload. Cooking is the responsibility of the women in this context. Thus, preparing a bread-based meal emerged as an added responsibility for woman. Switching to a *ruti-based* diet was even more challenging for working women, who usually had to bear a double day burden.

*Panta* or *thhandabhat* (rice left from last evening and fermented by soaking in water) was a common breakfast taken with onion and chili, not requiring any side dish. When fresh rice was served it was often eaten with mashed vegetables boiled along with rice, requiring minimal effort. Mashed vegetables are typically not eaten with *ruti*. Therefore, a separate side dish is required to go with the *ruti*.

“Is it possible to follow all the [dietary] recommendations? They suggest eating ruti in the morning. Now, how would I eat ruti without a side dish? If there is nothing to go with the ruti how would you get it (breakfast) ready quickly?”

-IDI 14, male

Inability of a woman to prepare a *ruti*-meal twice a day or a side dish to go with the bread pushed a few female diabetic patients to eat rice instead. As shown in Table 3, this was a challenge particularly for female compared to male patients (5 women vs. 1 man) despite the fact that in all households’ women were responsible for cooking. Thus, while some women reported preparing *ruti* for their own consumption as a challenge, only one man reported facing this challenge. This may suggest that women were more invested in care of the male patient with diabetes in the household than in self-care. Another possible explanation of lower reporting of this challenge by men may be that men were more able to purchase bread when home-made *ruti* was not available.

“I cannot gather the energy to make ruti after toiling the whole day. So, I eat rice [at dinner].”

-IDI 17, female

“[In order to avoid the hassle of making ruti] I skip dinner most of the time. Sometimes he (her husband) brings home [purchased] ruti. So, I have ruti [at dinner] one or two days [a week]. At times [when I skip dinner] I feel very hungry late at night and then I cannot help eating rice.”

-IDI 18, female

“I eat ruti and bhaji (fried vegetables) at breakfast. When this is not available at home, I maintain my diet … taking breakfast at the roadside restaurant.”

-IDI 4, male

This was also true for some women, who lived with adult son/s in an extended household, but not for the ones, who were part of a nuclear household. This difference between the two categories of women may be attributable to differences in their status. A mother-in-law in Bangladeshi society usually commands greater power and authority compared to a daughter-in-law. This was observed, however, only in a few cases.

Inadequate and irregular supply of cooking gas, a structural issue, also inhibited appropriate food preparation at home (Table 3). The gas supply was available very early in the morning at around 4 am for a brief period and once again at midnight. Thus, cooking was a major challenge and cooking additional items was extremely difficult.

“There is shortage of gas supply in the morning, which does not allow ruti preparation. Insufficient supply of cooking gas in the locality hardly supports rice cooking, let alone ruti making.”

-KII 3, male

“The doctor told me to eat plenty of vegetables replacing rice. If I can have a big bowl of vegetables. I’ll get rid of my hunger. …But I cannot cook vegetables due to lack of gas supply.”

-IDI 3, female

“It is a hassle to cook separately for myself after cooking for this large family. Recently, the gas supply has become irregular. This is why, I could not make ruti the last four to five days and so, ate rice.”

-IDI 22, female

Some patients (4 men and 4 women) reported facing challenges in eliminating sweets from their diet altogether (Table 3). Tea drinking is an essential part of socialising for men, in particular when they meet in tea shops. Some men found it difficult to avoid having tea with sugar or sugary condensed milk. Moreover, most men work outside the house and eat purchased snacks in between meals, which often contain sugar.

“While I plan to have half a cup of tea [with sugar] a day, I end up drinking more than seven cups of tea. Thus, (my) diabetes increases due to lack of control over tea drinking.”

-IDI 18, male

In a few households, all members tended to forgo sugar consumption for the sake of the male patient. Female patients did not have this advantage as they had to prepare sweet dishes for family members, making it harder for them to refrain from sweet consumption.

### Adherence to physical activity recommendations

Study participants recognised the importance of physical activity in controlling diabetes. However, only around a third of male and female patients reported walking regularly (Table 2). Younger women undertook physical exercise more than their older peers, disregarding harassment faced while doing so. Gender differences surfaced in the challenges faced in undertaking physical activity. Walking was difficult for many patients due to co-morbidities (9 men and 13 women). Twenty-two men and 24 women reported experiencing different challenges in walking (Table 3). Time constraint was a major deterrent to undertaking physical activity for both men (*n* = 12) and women (*n* = 17). For women, this was mainly due to household chores and care responsibilities (*n* = 11). Men reported time constraints in undertaking physical activity due to their workload related to income earning. Two out of five working women reported this constraint as well, while 12 out of 28 working men reported this as a barrier.

Findings suggest that structural constraints posed a major barrier to walking particularly for female patients (11 women vs. 2 men). Since the overwhelming majority of the women were not employed (85%), their busy schedule evolved around the house. Half of the women experienced time constraint in pursuing physical activity. Those who could manage to walk usually did so on the road adjacent to their houses. Thus, seven women and two men reported that they had to walk by the side of the road near heavy traffic, thereby compromising their safety. Four women reported a conflict between timing of cooking gas supply and a morning walk. COVID-19 was mentioned by a few male patients as interfering with physical activity.

One in three female patients suggested that social unacceptance of physical exercise by women and the potential associated criticism precluded women from undertaking physical activity other than walking. Sexual harassment and ridiculing were mentioned by four women. Men did not report these challenges.

### Differences between high- and low-performers in management of diabetes

Five men and four women regularly followed the recommendations made in 4–5 domains considered in this study and were included in the high-performing category (Table 6). In contrast, 6 men and 7 women did not follow the recommendations in 3 or more domains and were labelled as low-performers. The rest (19 men and 19 women) were treated as intermediates.

**Table 6:** Factors underlying performance status of diabetic patients by gender, Bauniabadh slum, Dhaka, 2021

Factor	High performing		Median		Low performing	
	Male (*n* = 5)	Female (*n* = 4)	Male (*n* = 19)	Female (*n* = 19)	Male (*n* = 6)	Female (*n* = 7)
Socio-economic status (SES)						
Poor	0	0	8	14	1	5
Non-poor	5	4	11	5	5	2
Marital status						
Married	5	2	19	19	6	6
Widow	0	2	0	0	0	1
Family size						
4 or less	3	3	5	6	1	2
5 or more	2	1	14	13	5	5
Lack of confidence in allopathic treatment/Reliance on kobiraji/ayurvedic/self-treatment						
Yes	0	0	0	1	3	1
No	5	4	19	18	3	6
Ever employed/empowered/agentic women						
Yes	-	3	-	12	-	6
No	-	1	-	7	-	1

All high-performers came from relatively better-off families. Family size varied between two and four for at least half of the members of this group. Those who had larger families in this category were financially stronger (e.g., substantially larger income and/or assets) and coped well with their family size. All the high-performing women except one were ever-employed. Two of the female high-performers were widows with extraordinary entrepreneurial skills. Both took good care of their own health, conscious of the fact that they had no one else to fall back on. There were two currently non-working high-performing women, who were valued in the family and were well looked after. Thus, it seems that a combination of better financial condition of the household, women’s employment, agency and status in the family contributed to the high performance in diabetes management in some women in this sample.

Regardless of performance category, male participants were the main breadwinners and thus, their health was a priority in the household. Resources were readily invested in the treatment and management of a man with diabetes, particularly in the high-performing group. Within the high-performing group the level of care taken by the family varied. For instance, in one case, family members were highly supportive of the needs of the man with diabetes. All the family members switched to a diabetic diet and the man was reminded to take his medicines, go for check-ups and follow recommendations for lifestyle modification. In order to encourage his physical activity, his wife accompanied him during his morning walk. In another case, the patient’s wife reminded him to take medicines, go for check-ups and follow recommended physical activity, but could not always prepare a separate diabetic diet for him.

Apart from sufficiently strong economic status of the household and family support, relevant connections seem to have contributed to making men high-performers. Thus, having a medical connection helped another high-performing man to get treatment advice whenever he needed it without any charge.

The low-performing group was not a mirror image of the high-performing group. Five out of six male and five out of seven female low-performers came from large families consisting of more than four household members. Interestingly, five out of six low-performing men came from non-poor households. It seems that their reliance on their own misperceptions and beliefs regarding treatment outweighed the potentially positive contribution of relatively better financial situation of these men in diabetes treatment and management. The female low-performers were quite different from their male peers. Only two out of seven female low-performers belonged to non-poor households. Financial challenges of the female low-performers in treatment and management of diabetes were compounded by large family size and lack of their own income.

While financial constraints feature as one of the major issues for female low-performers, negligence and inability to attach importance to diabetes treatment and management seemed to be a major challenge for at least two of the male low-performers. Mistrust and misperception of allopathic treatment, and confidence in self-medication prevented uptake of medical treatment by half of the male low-performers. The patient who had no confidence in allopathic treatment and never accessed it came from a family practicing *Kobiraji* medicine (i.e., traditional healing). Another man underwent allopathic treatment for 4 years and then he received an injection from a provider, which he believed had cured him of diabetes. So, he ceased seeking further treatment. Yet another man had full confidence in self-treatment based on his first-hand knowledge from his mother’s diabetes and whatever information he could gather from the internet.

Time constraint was a major challenge for following physical activity recommendations for all those in the low-performing group, but more so for females. While one-fifth of all the female sample faced this challenge, more than half of the female low-performers experienced it. Most of the women reporting inability to cut rice intake and inadequate gas supply interfering with bread-making belonged to the female low-performing group. Although six of the seven low-performing women were agentic their financial contraints and workload did not allow them to perform any better.

## Discussion

In this qualitative study, we identified that individual, household and structural factors posed significant gendered challenges to the management of diabetes. Men’s knowledge regarding diabetes management was based on a wider range of sources than for women, and included information derived from their higher mobility and access to the internet. Overall, these findings echo findings by Burner, Menchine, Taylor and Arora [12] in a study conducted among Latinas in the US.

Financial constraints in treatment-seeking, adhering to medicine and diet-related recommendations were much larger issues for women than for men. This was not only because most women did not earn, but also due to a lack of access for women to household resources and women’s altruism, expressed in prioritising household and family members’ needs over their own treatment. It is important to point out that women’s financial constraints are not only typical for a slum population from a relatively low-income country, but also for female patients with diabetes in the US [14]. This is because structural issues, unequal intrahousehold resource allocation and social norms disadvantage women. This, underlines the importance of paying attention to gender in diabetes management regardless of the relative wealth of the nation.

Notwithstanding the greater number of challenges experienced by women, their performance in medical treatment seeking was not much different from that of the men. This may be explained by women’s use of a broader range of positive coping strategies compared to men in dealing with their greater and deeper financial problems. This finding lends support to the finding by DeCoster & Cummings [42] in a US sample. Relatively greater reservation against seeking financial support from others in treatment of diabetes among men compared to women may be due to lower financial needs and an urge to uphold the masculine image of a successful or adequate income earner. The fact that fewer men than women used skipping or stopping medical treatment as a strategy for coping with financial constraints without accessing financial support from others may indicate lower financial stress or greater access of men to household financial resources. Although women’s use of negative coping strategies also exceeded that of the men, it seems that the positive coping strategies employed by women outweigh the negative ones.

A cluster of exclusively female, not purely financial issues surfaced in medical treatment-seeking. One of them was distance to the provider, which interacted with women’s lack of agency to go to places on their own; women’s financial problems; and time constraints (due to traffic congestion). These findings echo the findings from South Asia by Bhojani et al. [16]. Issues around women’s lack of mobility had been successfully addressed in the Family Planning programme of Bangladesh by implementing a doorstep service delivery strategy in the rural areas several decades ago [43]. The possibility of such interventions in slums needs to be considered. While trying to set up services closer to home for slum dwellers, improvement of the quality of services would also deserve careful attention.

The predominant energy food source for a Bengali is rice [44]. Three rice-meals are often preferred in low-income households of Bangladesh, and a huge amount of rice is usually consumed at each meal in order to fulfil energy needs [44]. Thus, dietary recommendations to reduce rice intake and complement rice with *ruti* and vegetables were a challenge for both male and female patients. Moreover, this recommendation had adverse implications for women’s workload. Overall, replacing rice with *ruti* was more of a challenge for female than male patients as woman often preferred either to skip a meal or to eat a rice meal rather than have to prepare *ruti* for herself after completing all other household chores and care work. In contrast, male patients (and a couple of mothers-in-law) were able to eat purchased *ruti*, if home-made *ruti* was not available. Lack of cooking gas supply, a structural issue also contributed to the inability of a woman to prepare bread or an additional vegetable dish.

Our findings clearly show how seemingly neutral structural factors such as public space and cooking gas supply are experienced differently by men and women carrying implications for diabetes management. Although both sexes referred to challenges in undertaking physical activity, women grappled with a greater number. Almost half of the women mentioned structural issues impeding walking. Both men and women mentioned constraints of space for walking. Moreover, time constraints due to household responsibilities made it easier for them to walk on the nearest (usually crowded) roads compromising their safety. These findings substantiate findings by Giles-Corti and Donovan [28].

Previous studies elsewhere suggest that altruism on the part of women often does not allow them to seek medical treatment and adhere to recommended medication and lifestyle changes [45]. Women’s altruism was expressed in: unwillingness to disclose their treatment needs; prioritising household chores over self-care; and reluctance to change lifestyle which might affect the ability to accommodate the needs and comfort of others [17–20]. The current study conducted in a different context echoes these findings, demonstrating commonality of attitudes and behaviours among women across different settings.

Women’s unpaid care work, lower or no income altogether, limited decision-making power over household resources and their own healthcare and lack of agency, converge to create significant barriers to accessing health care and management of disease. Women’s access to care is further constrained by health systems and the broader political economy, which mirror and reinforce restrictive gender norms, unequal power relations, and systematic discrimination.

Factors underlying high and low performance varied by gender. Our findings suggest that strong financial status of the household, relevant connections and overwhelming support from the family underlie high-performance of men. Strong financial status of the household is a necessary, but not adequate condition to enable high-performance among women. Women’s economic empowerment, agency, voice and power seem to be key to facilitating high-performance.

Findings show that the low-performers are not mirror images of high-performers. Thus, most of the men in the low-performing category actually came from financially strong households. It seems that lack of confidence in allopathic treatment and misperception about treatment as a whole, contribute to men’s low-performing status. Negligence and inability of men to attach importance to diabetes management were other contributors to their low performance. As previously reported [46, 47] these are essentially manifestations of norms of masculinity in a patriarchal society that condone and promote men’s stoicism and self-reliance thereby hampering treatment seeking and diabetes management. Despite relatively higher agency, financial constraints and workload inhibited better performance of the female low-performers.

To our knowledge this is the first study addressing the different challenges and needs of women and men with type 2 diabetes living in low income under-served urban areas in Bangladesh, and the first attempt to conduct an analysis of high- and low-performance in management of diabetes among women and men. Strengths of the study include a population-based sampling frame, rigour in data coding and analysis, and the novelty of the findings. However, caution must be applied in interpreting these results due to some study limitations. First, this study was dependent on self-report, which may be affected by social desirability bias, which may in turn be differential by sex and socio-economic position. The male and female samples could not be matched exactly which reflects gender differences in this society and in the slum population, in particular. Due to power hierarchy between the genders, females are subject to numerous discriminations at multiple layers of the society and family. Thus, the female sample had lower education, very low employment, and was poorer than the male sample. In this context, over controlling could potentially mask true gender differences that influence our outcomes of interest.

Due to high COVID-19 infection rates, in-person interviews, which are believed to yield higher quality data could not be undertaken. The pandemic combined with low access to technology also made it impossible to conduct Focus Group Discussions (FGDs), which could help to better triangulate the data. We did, however, triangulate the data using IDIs and KIIs. The data reached saturation fairly quickly. Thus, it can be assumed that rigorous results were obtained in the absence of face-to-face interviews and FGDs.

This topic could also benefit from mixed-methods research. A follow up quantitative study could be undertaken to validate the results.

The findings highlight the importance of tailoring diabetes policies and programmes according to different gendered needs. In order to facilitate treatment-seeking and adherence to prescribed medication in this low-income population exhibiting pronounced gender hierarchy, it is important for the government to consider introducing subsidised consultation charges and reduced price of medicines for the poor in general, and for women in particular, who have lower access to, and control over, resources compared to men.

An analysis of unintended gender consequences of dietary recommendations (e.g., *ruti* vs. rice preparation) highlights an important issue of how well-intentioned health guidance can inadvertently exacerbate gender inequalities. This insight should inform the health practitioners to use more gender-sensitive approaches to dietary recommendations. Insufficient and inadequate cooking gas supply make it more challenging for women to adhere to dietary and physical activity-related recommendations. This important structural issue, which overlaps with gender, demands attention from the government. Policymakers and programme implementers may also consider popularising foods that are low in starch content but easier to prepare. An example of such food may be *chhatu*, a pulse or corn-based powder, which can be made into a paste by mixing it with water or milk.

Although general awareness regarding diabetes management is quite high in this population, there remains a small group of patients whose lack of confidence in, and misperceptions about, allopathic treatment need to be addressed.

Urban planning needs to consider the provision of open and safe spaces (e.g., with lights) for physical activity. The government needs to ensure that these plans are implemented and the spaces remain functional and continue to be devoted to physical activity.

Female patients need to be empowered to access resources and to increase their agency to claim their rights and to deal with the outside world. Promoting employment opportunities for women may help improve diabetes treatment and management. Awareness needs to be promoted among female patients regarding harms involved in negative coping with financial constraints in diabetes treatment. Practitioners need greater awareness of gender-patterned constraints to optimal management of diabetes when assessing patients and making recommendations.

## Conclusions

This paper presents an in-depth exploration of how differences in gender roles, gendered access to, and control over, resources, and social norms contribute to treatment and management of diabetes in an urban slum of Bangladesh. Although gender differences are well documented in many patriarchal societies their impact on treatment and management of diabetes has rarely been studied. Our findings show that multilevel factors operating at the individual, household, and structural levels contribute to gender differentiated treatment and management of diabetes. Novel are our findings regarding the factors underlying high-performance among men and women. Financial strength, relevant connections and support from the family underlie high-performance by men in the management of diabetes. Household financial status is a necessary, but not sufficient condition for high-performance among women. Women’s economic empowerment, agency, voice and power seem to be key for their high-performance in managing diabetes.

Our findings highlight the importance of tailoring programs and policies for diabetes to the differential needs of women and men. In order to tackle diabetes effectively, it is essential for programs and policies to not only empower individual patients, but also to address gender hierarchical social norms and structures in context, where inequitable gender norms remain salient.

## Supplementary Information

Supplementary Material 1.

## Data Availability

Data is provided within the manuscript and supplementary information files.

## Abbreviations

BELIEVE: Bangladesh longitudinal investigation of emerging vascular and nonvascular events
BSMMU: Bangabandhu Sheikh Mujib Medical University
icddr,b: International Centre for Diarrhoeal Disease Research, Bangladesh
IDI: In-depth interview
IDIs: In-depth interviews
KII: Key informant interview
KIIs: Key informant interviews
NCDs: Non-communicable diseases
NHF: National Heart Foundation
T2D: Type 2 diabetes
