# Supplementary material for: Gender differences in type 2 diabetes treatment and management: a qualitative study in an urban slum population from Dhaka, Bangladesh
Source: Int J Equity Health. 2025 Sep 30;24:243. doi: 10.1186/s12939-025-02611-2 (PMC12487208; doi:10.1186/s12939-025-02611-2)
Supplement: Supplementary file 1 — Supplementary Material 1. [file 12939_2025_2611_MOESM1_ESM.pdf]

Additional File 1: In-depth interview guide and key informant interview guide

In-depth Interview Guide for Telephone-based Data Collection

Background Information of participant:
Age:
Marital status:
Occupation: Get detailed notes before and after CORONA. Probe: what kind of physical activity it involves.
Highest Level of Education:
Family type:
Number of family members before and after CORONA:
Number of children before and after CORONA:
Relationship with different family members:
Household head before and after CORONA:
Income before and after CORONA (Probe: Household income and individual income - what kind of changes took place after CORONA):
SES before and after CORONA:

Guide:

Part 1: Perceptions about diabetes; Diagnosis, management and treatment of diabetes
Note for the interviewer: Tell the informant--You know, we are passing a critical period and facing different situations due to The Corona pandemic this year. It has affected the life of different people in different ways. For answering the next questions I would request you to describe your perceptions about diabetes and the diagnosis of your diabetes. Besides I want you to tell me about the management and treatment you usually followed for diabetes before Corona. Following it you will tell me if things changed due to Corona.

Domain/Focus area	Questions and probes
**Perceptions about diabetes**	1. Please tell me about your perceptions regarding this disease (Probe: what kind of disease it is, why it happens, how you can get this disease, what are the consequences of this disease? what measures can a person take to prevent this disease? What are the sources of treatment for diabetes?<br><br>How did you get these ideas/information about diabetes? Did you know about this disease before you became diabetic? How? Please tell me in detail.
**Diagnosis, management and treatment of diabetes**	2. For how long are you suffering from diabetes? How it was diagnosed?<br>3. How long did it take you to seek treatment after you were diagnosed? If there was a gap, why was that (lack of money, permission, chaperon)?<br>4. Who did you seek treatment of diabetes from? How did you get his/her information?<br>5. Is your diabetes in control most of the time? If not, why not? If yes, how do you manage to keep it in control? How did you get these ideas/information?<br>6. How frequently do you have follow up visits with a provider usually? Who do you usually go for follow up? If he/she goes irregularly or after a long time, what are the reasons? Please tell me about any change that occurred in case of your follow up visits due to Corona.<br>7. What kind of diabetes treatment are you on usually? (Probe: Medication, insulin, diet control, physical activity). Is there any change due to Corona?<br>8. If the informant is advised to take prescribed medicines, do you take them regularly? If not, why? Is there any change due to Corona?<br>9. How frequently do you have diabetes tests usually? If not monthly, what are the reasons (financial, lack of time, lack of initiative, lack of support from home)? Please tell me about any change that occurred in case of your diabetes tests due to Corona.<br>10. When seeking necessary treatments and taking medications as a diabetic, which

	factors usually act as facilitators or barriers for you? (Probe: SES, education, gender roles and position at home, responsibilities and duties at home/at workplace, decision making power at home/at workplace or lack thereof, ownership of familial wealth or lack thereof, familial and social points of view and expectations, self-confidence or lack of confidence, less/excessive work pressure, lack of security, behavior or harassment of healthcare providers at healthcare center, insufficient male/female physicians at healthcare center, etc.).<br><br>How, do you think, these barriers can be removed?<br><br>Ask the informant: When seeking necessary treatments and taking medications as a diabetic, which factors acts as facilitators or barriers for you during Corona? How, do you think, these barriers can be removed?

<u>Part 2: Food</u>

Note for the interviewer: Tell the informant--Now I am going to ask you some questions about your food-habits. I would request you to answer me about your usual food-habits before Corona. Following it you will tell me if things changed due to Corona.

**Domain/Focus area**	**Questions and probes**
**Food**	11. What dietary advice did you receive from the provider for keeping diabetes in control (avoid oil, fat and red meat such as beef or lamb in diet)? Are you able to follow these? Why or why not?<br><br>12. What kind of food do you usually eat (e.g., carbohydrates/vegetables/red meat/other meat/fruits)? Please tell me about any change that occurred due to Corona.<br><br>13. Do you usually eat your meals last?<br><br>Do you eat what you want to and how much you wished to or are there any constraints? (Probe: proportion of: carbohydrates/vegetables/red meat/other meat/fruits; leftovers; fresh or stale food? Please tell me about any change that occurred due to Corona.<br><br>14. What do you think are the facilitators to bring changes in your food habits for complying with the health providers' instructions (Probe: SES, education, gender roles and positions at home/at workplace, responsibilities and duties at home/at workplace, decision making power at home/at workplace or lack thereof, ownership of familial wealth or lack thereof, familial and social points of view and expectations, situations related to Corona, etc.)?<br>What do you think are the barriers to bring changes in your food habits for complying with the health providers' instructions? How, do you think, these barriers can be removed?

<u>Part 3: Physical activity</u>

Note for the interviewer: Tell the informant--Now I am going to ask you some questions about your physical activities. I would request you to answer me about your usual physical activities (such as walking/running outside home, taking exercise, etc) before Corona. Following it you will tell me if things changed due to Corona.

**Domain/Focus area**	**Questions and probes**
**Physical activities**	15. What lifestyle advice did you receive from the provider for keeping diabetes in control (walking, taking exercise, keeping stress level low)?<br><br>Are you able to follow these? Why or why not? (Probe: Less/excessive pressure of work-at home/at workplace, freedom/lack of mobility, less/excessive social restrictions/social , adequate security/lack of security, adequate space/lack of space, any situation related to Corona, etc.).<br><br>16. What kind of physical activities do you usually follow (e.g., walk around rapidly/run

	outside the home, other exercise/work-out, any sort of game/sports, etc)?<br>How do you make the time and get the opportunity? Please tell me about any change that occurred due to Corona.<br><br>17. What do you think are the facilitators to bring changes in your physical activity level (walking/running/exercising/sport-playing) (Probe: SES, education, gender roles and positions at home/at workplace, responsibilities and duties at home/at workplace, decision making power at home/at workplace or lack thereof, ownership of familial wealth or lack thereof, familial and social points of view and expectations, any situation related to Corona, etc.)?<br><br>What do you think are the barriers to bring changes in in your physical activity level? How, do you think, these barriers can be removed?

<u>Part 4: Managing mental stress</u>
Note for the interviewer: Tell the informant-- ***Every person has dreams and expectations from life and from people around him/her. Some of these dreams and expectations materialize, while others do not, which may cause frustration and stress. Further, as the saying goes, "plates in a basket will rattle". People living together may agree and disagree on many topics leading to various positive and negative experiences. Sometimes these experiences create stress.***
I would request you to answer me about your usual experience of mental stress before Corona and how did you manage it usually before Corona. Following it you will tell me if things changed due to Corona.

Domain/Focus area	Questions and probes
**Managing mental stress**	18. Which situations at home and work or in your life do you think, usually create mental stress for you? (Probe: Gender roles and position at home/at workplace, responsibilities and duties at home/at workplace, conflict with household members; derogatory treatment at home, work or outside; etc.) Please tell me how things changed due to Corona. How do you think you can overcome this?<br><br>19. What do you usually do to reduce your mental stress in various situations? Please tell me how things changed due to Corona.<br><br>Do you notice any difference in your coping to mental stress since you have become a diabetic?<br><br>20. ***Ask only female respondents:*** Which specific situations create mental stress for you, especially because you are a woman? (Probe: not being able to participate in decision-making, restrictions to mobility, restrictions to communication, experience of violence-at home/outside home/at workplace, etc.) Please tell me in details of these situations. How do you think you can overcome this?<br>What do you do to reduce your mental stress in various situations? Please tell me how things changed due to Corona.<br>Do you notice any difference in your coping to mental stress since you have become a diabetic, especially because you are a woman? Please tell me in details.

<u>Part 5: Use of tobacco and othe harmful items</u>
Note for the interviewer: Tell the informant-- ***Now I will ask you about some specific habits. Please answer if you feel comfortable. Otherwise, you can skip these questions.***

Domain/Focus area	Questions and probes
**Use of tobacco and othe harmful items**	21. Have you ever consumed any betel leaf and related products (*paan, chun, shupari, etc.*)?<br>*If yes, then:* Please tell us more about this habit. (Probe: How long have you been using this, how frequently do you use this. Why did you use this?) Do you still engage in this

	habit? Is there any change in your habit due to Corona? To date, have you ever faced any health problems because of this habit? What did you do then?<br><br>22. Have you ever consumed any tobacco products including *beedi*, cigarette, *jorda* (flavored chewing tobacco flakes), *gul* (powdered tobacco), etc.)?<br>*If yes, then:* Please tell us more about this habit. (Probe: How long have you been using this, how frequently do you use this. Why did you use this?) Do you still engage in this habit? Is there any change in your habit due to Corona? To date, have you ever faced any health problems because of this habit? What did you do then?<br><br>23. Do you want to tell me about any other items which you use and think that it is not good for managing your diabetes?<br>*If yes, then:* Please tell us more about this habit. (Probe: How long have you been using this, how frequently do you use this. Why did you use this?) Do you still engage in this habit? Is there any change in your habit due to Corona? To date, have you ever faced any health problems because of this habit? What did you do then?

Part 6: Conclusion

24. Do you have anything else to say related to what we just discussed?

I know diabetes is a difficult disease to cope with. I hope you'll be able to overcome the challenges in keeping it in control. Thank you for your time.

Key Informant Interview Guide for Telephone-based Data Collection

Background Information of participant:
Age:
Occupation:
Highest level of education:
Informant’s social position and involvement in the community:

Guide:

Part 1: Perceptions about diabetes and prevelance in the area
Note for the interviewer: Tell the informant-- For answering the next questions I would request you to describe the perceptions about diabetes and prevelance of this disease in your area.

Domain/Focus area	Questions and probes
**Perceptions about diabetes and pervasiveness of diabetes in the area**	1. What do most of the men and women in your area understand by diabetes (Probe: what kind of disease it is, why it happens, how one can get this disease, how it can be treated, etc)? How did they get these understandings?<br><br>Does this disease have any specific/special name in your area? If so, then what are these names?<br><br>2. Is diabetes widespread in your area? Is it common both among men and women?<br>What are the usual characteristics of diabetic men and women (Probe: age, SES, education, obesity, sedentary lifestyle, food habits, etc)?

Part 2: Treatment of diabetes and compliance by gender
Note for the interviewer: Tell the informant--You know, we are passing a critical period and facing different situations due to The Corona pandemic this year. It has affected the life of different people in different ways. For answering the next questions I would request you to describe the treatment which diabetic men and women in your area usually followed before Corona. Following it you will tell me if things changed due to Corona.

Domain/Focus area	Questions and probes
**Treatment of diabetes and compliance by gender**	3. What are the treatment options usually available for diabetic patients in your area? Who do most male and female diabetic patients go? (Probe: How things changed for the male and female diabetic patients in terms of seeking help from the health care provider due to Corona?)<br><br>4. What kind of treatment are men and women usually on? (Probe: How things changed for the male and female diabetic patients in terms of having treatment due to Corona?)<br><br>5. Among the male and female diabetic patients in your area, whom do you find more regular in checking diabetes – male patients or female patients? How regular are they and why? If not, why not (Probe: avoidance, lack of money, lack of initiative, lack of support, any situation related to Corona,)?<br><br>How things changed for the male and female diabetic patients in terms of checking diabetes due to Corona?<br><br>6. Among the male and female diabetic patients in your area, whom do you find more compliant in taking medicines and insulin as advised? How regular are they and why? If not, why not (Probe: avoidance, lack of money, lack of initiative, lack of support, any situation related to Corona,)?<br><br>How things changed for the male and female diabetic patients in terms of taking medicines and insulin due to Corona?

<u>Part 3: Physical activity</u>

Note for the interviewer: Tell the informant--for answering the next questions, I would request you to describe the physical activities of diabetic men and women in your area which they usually followed (such as walking/running outside home, doing exercises, etc) in controlling diabetes before Corona. Following it you will tell me if things changed due to Corona.

Domain/Focus area	Questions and probes
**Physical activity**	7. Among the male and female diabetic patients in your area, whom do you usually find more engaged in any kind of exercise/sports, like: walking rapidly/ running outside home or any other kind of exercise/sports?<br>- How frequently men and women do so,<br>- how long men and women usually exercise/play sports,<br>- where do men and women exercise/play and<br>- why men and women exercise/play there.<br>Please tell me about any change occurred due to Corona.<br>8. Which kind of diabetic men and women in your area usually exercise/play games? Please tell me about any change occurred due to Corona.<br>Which kind of men and women do not do so? Why? (Probe: excess pressure of work at home/at workplace, barriers to mobility, social restrictions, lack of security, etc.)

<u>Part 4: Diet</u>

Note for the interviewer: Tell the informant-- for this part of discussion, I would request you to answer me about the diet of diabetic men and women which they usually followed in controlling diabetes before Corona. Following it you will tell me if things changed due to Corona.

Domain/Focus area	Questions and probes
**Diet**	9. Among the male and female diabetic patients in your area, whom do you usually find more complying to maintain proper diet (e.g. avoid sugar and sweet foods, cut on carbohydrate rich diet, avoid oil and fat, and red meat such as beef and lamb) for controlling their diabetes? Why?<br>How do the men and women diabetic patients maintain proper diet?<br>What, do you think, are the facilitators to bring changes in the diet-habit of diabetic men and women?<br>What are the barriers (Probe: Hard to change habit/preference, cannot afford, no support, any situation related to Corona, etc)? How, do you think, these barriers can be removed?<br>10. Which kind of diabetic men and women of your area usually can maintain proper diet for controlling their diabetes? Please tell me about any change occurred due to Corona.<br>Which kind of men and women cannot do so? Why? (Probe: excess pressure of work at home/at workplace, hard to change habit/preference, cannot afford, no support, etc.)

Part 5: Managing mental stress

Note for the interviewer: Tell the informant-- *People have various positive and negative experiences in their lives. Sometimes these experiences create mental stress on people.* For answering next questions, I would request you to describe me about the mental stress of the diabetic men and women in your area usually suffers from and how they usued to managed their mental stress before Corona. Following it you will tell me if things changed due to Corona.

**Domain/Focus area**	**Questions and probes**
**Managing mental stress**	11. Which situations at home and work, or outside do you think, usually create mental stress for the men and women in your area? (Probe: Gender roles and position at home/at work place, responsibilities and duties at home/workplace, lack of communication between spouses; lack of communication with relatives, neighbours, peers; lack of communication between co-workers, home setting/work environment etc.). Please tell me in details regarding various situations.<br><br>How things changed due to Corona?<br><br>12. What do most of the men and women in your area usually do to reduce their mental stress in various situations? How things changed due to Corona?<br><br>13. Do you observe any difference in coping with mental stress among men and women before and after they became diabetic? How do you think men and women can overcome mental stress?<br><br>14. ***Ask next questions separately for female community members:*** Which specific situations usually create mental stress especially because they are women? (Probe: not being able to participate in decision-making, restrictions to mobility, restrictions to communication, experience of violence, etc.) Please tell me in details regarding various situations.<br>How things changed due to Corona?<br><br>What do most of the women in your area do to reduce their mental stress in these situations? How things changed due to Corona?<br><br>Do you observe any difference in coping with mental stress among women before and after they became diabetic? How do you think women can overcome this kind of mental stress?

Part 6: Use of tobacco and other harmful items

Note for the interviewer: Tell the informant-- *Now I will ask you about some specific habits of the men and women in your area. Please answer if you feel comfortable. Otherwise, you can skip these questions.*

**Domain/Focus area**	**Questions and probes**
**Use of tobacco and other harmful items**	15. How pervasive is the use of various tobacco products in your area? [Including *beedi*, cigarette, *jorda* (flavored chewing tobacco flakes), *gul* (powdered tobacco), etc.] Who use which type of product mostly (is there difference in product usage by demographic section)? Is there any change due to Corona? What kind of health problems do they face because of these habits? Are there any differences between men and women in the usage of these products? What are the differences? Do the men and women those are diabetic use these products?<br><br>16. How pervasive is the use of various betel leaf and related products (*shupari, chun*) in your area? Who use which type of product mostly (is there difference in product usage by demographic section)? Is there any change due to Corona? What kind of health problems do they face because of these habits? Are there any differences between men and women in the usage of these products? What are the differences? Do the men and women those are diabetic use these products?

	17. Do you want to tell me about any other harmful habits of men and women in your area which is not good for managing diabetes?<br>*If yes, then:* Please tell me more about these habits and problems men and women diabetic patients experience for these habits.

Part 7: Conclusion

18. Do you have anything else to say related to what we just discussed?

Thank you for your time
